# How physical techniques improve the transdermal permeation of therapeutics: A review

**DOI:** 10.1097/MD.0000000000029314

**Published:** 2022-06-30

**Authors:** Yan Gao, Lina Du, Qian Li, Qi Li, Lin Zhu, Meiyan Yang, Xiu Wang, Bonian Zhao, Shan Ma

**Affiliations:** a Institute of Pharmacy, Shandong University of Traditional Chinese Medicine, Jinan, China; b Department of Pharmaceutical Sciences, Beijing Institute of Radiation Medicine, Beijing, China; c State Key Laboratory of Toxicology and Medical Countermeasures, Beijing Institute of Pharmacology and Toxicology, Beijing, China; d School of Medicine, Bengbu Medical University, Bengbu, China; e Shandong Co-Innovation Center of Classic TCM Formula, Shandong University of Traditional Chinese Medicine, Jinan, China.

**Keywords:** electroporation, iontophoresis, laser, magnetophoresis, microneedles, microwaves

## Abstract

Transdermal delivery is very important in pharmaceutics. However, the barrier function of the stratum corneum hinders drugs absorption. How to improve transdermal delivery efficiency is a hot topic. The key advantages of physical technologies are their wide application for the delivery of previously nonappropriate transdermal drugs, such as proteins, peptides, and hydrophilic drugs. Based on the improved permeation of drugs delivered via multiple physical techniques, many more diseases may be treated, and transdermal vaccinations become possible. However, their wider application depends on the related convenient and portable devices. Combined products comprising medicine and devices represent future commercial directions of artificial intelligence and 3D printing.

**Methods:**

A comprehensive search about transdermal delivery assisted by physical techniques has been carried out on Web of Science, EMBASE database, PubMed, Wanfang Database, China National Knowledge Infrastructure, and Cochrane Library. The search identified and retrieved the study describing multiple physical technologies to promote transdermal penetration.

**Results:**

Physical technologies, including microneedles, lasers, iontophoresis, sonophoresis, electroporation, magnetophoresis, and microwaves, are summarized and compared. The characteristics, mechanism, advantages and disadvantages of physical techniques are clarified. The individual or combined applicable examples of physical techniques to improve transdermal delivery are summarized.

**Conclusion:**

This review will provide more useful guidance for efficient transdermal delivery. More therapeutic agents by transdermal routes become possible with the assistance of various physical techniques.

## 1. Introduction

Transdermal delivery has clear advantages over injection and oral routes in terms of increasing patient compliance and avoiding first-pass metabolism. The skin, as one of the largest organs in the human body, has a surface area close to 1–2 m^2^ available for drug delivery. Moreover, the application of a patch-like device to the skin surface can be controlled manually, for example, by removal, replacement, or repositioning. The major advantages of a transdermal drug delivery system (TDDS) include:

1. Patients can self-administer at home;
2. The continuous release of the drug can ensure that the drug concentration in the body remains at a stable level;
3. Avoids first-pass hepatic metabolism and enzymatic degradation in the gastrointestinal tract and also avoids gastrointestinal irritation;
4. Less frequent dosing improves patient compliance;
5. Alternative to other routes of administration;
6. Dose delivery unaffected by vomiting or diarrhea;
7. The drug release can be stopped at any time by removing the TDDS.

Previously, TDDSs developed slowly because of the many limitations of drugs. Molecular weight (MW) is the major limiting factor. In general, drugs with an MW < 500 Da^[1]^ are the best choice for transdermal permeability. However, increasing numbers of bioactive macromolecules have been developed or identified with the rapid development of genomics and biotechnology. Improving the transdermal permeability of bioactive macromolecules is important.^[2]^

## 2. Skin structure

TDDSs can achieve systemic therapeutic effects through topical administration on the skin. Consequently, it is critical to understand the barrier function, biochemical properties, and structure of skin, and the rate at which drugs enter the body through the skin.

The skin is generally divided into 3 layers: The stratum corneum (SC), the epidermis, and the dermis, which are penetrated by sweat glands and hair follicles. The SC is located on the outside and is composed of dead cells. Therefore, the epidermis without the SC is often referred to as the viable epidermis. The SC is the most important rate-limiting barrier for most molecules during transdermal penetration. From a dry to a wet state, the thickness of the SC swells from 10–15 to 40 μm. Keratin-rich corneocytes in the SC structure are often compared with bricks embedded in a lipid-rich matrix (the “mortar”). This SC arrangement has about 15–20 layers.

The epidermis is located on the dermis and is a superficial structure of the skin. The dermis mainly comprises fibroblasts, and the fibers and stroma they produce, and has nerves, lymphatic vessels, blood vessels, skin appendages, and other cellular components. The epidermal layer is located on the outermost layer of the skin and is the protective layer of the body skin, with an average thickness of about 0.1 mm, which can protect the skin from sun, microorganisms, and other external environments.^[3,4]^ The extracellular matrix lipid is different in many ways.^[5]^

1. It is the only continuous phase (and diffusion pathway) from the skin surface to the SC base.
2. In contrast to other biofilms, it does not contain phospholipids.
3. SC lipids exhibit a multilayered sheet shape in the absence of polar bilayer-forming lipids.
4. Compared with the usual liquid crystalline membrane, the highly ordered, intersecting configuration, and gel-phase membrane domains are more favorable for its formation under conditions of saturated long-chain hydrocarbon tails.

## 3. Major Transdermal Routes

The multiple advantages of TDDSs have made them a research hot spot, and many commercial formulations are currently available (Table 1).

**Table 1:** Drugs approved by FDA for transdermal administration.

Drugs	Company	Indication	Approval year
Rivastigmine	Novartis	Dementia	2007
Rotigotine	UCB, Inc.	Parkinson disease	2007
Granisetron	Prostrakan, Inc.	Chemotherapy-induced nausea and vomiting	2008
Oxybutynin	Watson	Urinary incontinence	2009
Chloride	Allergan	Overactive bladder	2009
Buprenorphine	Purdue Pharma L. P	Pain and opioid dependence	2010
Scopolamine	Glaxosmithkline CON	Motion thickness	1979
Nitroglycerin	Hospira	Angina pectori	1981
Clonidine	Boehringer Ingelheim	Hype tension	1984
Estradio	Novartis	Menopausal symptoms	1986
Fentanyl	JANSSEN PHARMS	Chronic pain	1990
Nicotine	Sanofi-Aventis US	Smoking cessation	1991
Testosterone	Alza Pharmaceuticals	Testosterone deficiency	1993
Estradiol/norethisterone	Parke Davis	Menopausal symptoms	1996
Ethinylestradiol/norelgestromin	JANSSEN PHARMS	Contraception	2001
Estradiol/levonorgestrel	BAYER HLTHCARE	Menopausal symptoms	2003
Lidocaine with tetracaine		Local dermal analgesia	2004
Methylphenidate	NOVEN PHARMS INC.	Hyperactivity disorder	2006
Selegiline	Somerset	Depressive disorder	2006
Diclofenac epolamine	Inst. Biochem.	Acute pain	2007
Capsaicin		Neuropathy pain	2009
Influenza-virus vaccine		Influenza virus	2011

FDA = Food and Drug Administration.

There are 3 critical methods by which drugs can pass through an intact SC: via the intercellular lipid domains, the skin appendages, or the transcellular route. The transdermal flux of a drug through a specific pathway is determined by the physicochemical properties of the molecule.^[6]^

### 3.1. The appendageal route

The transdermal routes include permeation through the sweat glands and across the hair follicles with their associated sebaceous glands. Skin appendages are an important continuous channel across the SC barrier. The opening diameter and follicular number volume are important in drug delivery. The forehead, as the follicular infundibula, occupies 13.7 mm^2^/cm^2^ (approximately 0.1%) of SC of forearm skin.^[5]^

### 3.2. Transcellular route

Keratinocytes containing highly hydrated keratin can increase the permeability of hydrophilic drugs that cross the skin through the transcellular route. The transcellular pathway requires that drugs can distribute and diffuse into the keratin bricks and the intercellular lipids.

### 3.3. Intercellular route

The intercellular route is a challenging pathway because it requires the drug to diffuse through the continuous lipid matrix.

1. Compared with the relatively direct pathway of the transcellular pathway, the interdigitating nature of corneocytes results in a tortuous pathway for intercellular drug permeation.
2. Drugs need to be sequentially assigned to the water and lipid domains of the intercellular domain and repeatedly diffused through them. This approach is only suitable for transdermal delivery of uncharged small molecules.

## 4. Physical Techniques To Improve Transdermal Delivery

There are a number of solutions to overcome the barrier function of the SC, such as chemical enhancers, novel formulations, and physical improvement techniques. Chemical penetrators have been developed to improve drug dermal penetration, such as azone^[7]^ and cyclodextrins.^[8]^ However, besides their limited transdermal penetration enhancement efficiency, they are also associated with side effects, such as rash, irritation, and hypersensitivity. Thus, novel formulations have been developed to improve transdermal absorption, including nanoparticles,^[9]^ liposomes,^[10,11]^ transferosomes,^[12]^ microemulsion,^[13,14]^ hydrogels,^[15,16]^ invasomes,^[17]^ and ethomes.^[18]^

Physical penetration technologies have developed quickly along with the emergence of technologies, such as microelectromechanical systems, artificial intelligence, microneedles (MNs),^[19,20]^ sonophoresis,^[21,22]^ iontophoresis,^[23,24]^ electroporation (EP),^[25,26]^ microwave,^[27]^ magnetophoresis,^[28]^ and lasers.^[29]^ They can change the skin surface structure directly and reversibly to enhance transdermal absorption.^[30–32]^ Moreover, these technologies are safe and efficient, show high bioavailability, and have been used widely, making them a better choice to promote the transdermal absorption of drugs (Table 2).^[33]^

**Table 2:** Companies developing and commercializing skin permeability technologies.

Technologies	Company	Product	Mechanism
Low-frequency sonophoresis/Sensing	Echo Therapeutics, Inc.	Symphony Continuous	Glucose monitoring by interstitial fluid after skin
		Glucose Monitor	Permeability
Microneedles	Becton Dickinson/Sanofi Pasteur	Intanza	Prefillable injection system utilizing the microneedle for delivery of vaccine against seasonal influenza
	Corium International, Inc.	MicroCor	Dissolvable microneedle device
	NanoPassTechnologies, Ltd.	MicronJet	Hollow microneedle device
	Seventh Sense Biosystems, Inc	TAP 20C	Microneedle penetration of the skin followed by application of vacuum for blood extraction
	TheraJect	TheraJect Patch	Dissolvable microneedle device
	Valeritas, Inc.	Micro-Trans	Microneedle device
	Vaxxas, Inc.	The Nanopatch	Vaccine-coated microneedle device
	ZosanoPharma	ZP Patch	Drug-coated microneedle patch
Electrical techniques	Ichor Medical Systems	TriGrid Delivery System	Electroporation platform for vaccination
	Inovio Pharmaceuticals, Inc.	Cellectr	Electroporation platform for vaccination
	NuPathe, Inc.	Zecuit	Iontophoresis-facilitated delivery of sumatriptan for acute treatment of migraines
	OncoSec Medical, Inc.	OncoSec Medical System	Electroporation platform for vaccination
	NB Therapeutics	Iontophoresis Platform	Iontophoresis-facilitated delivery of terbinafine HCl for the treatment of toenail fungus

### 4.1. Microneedles

**Figure 1.**
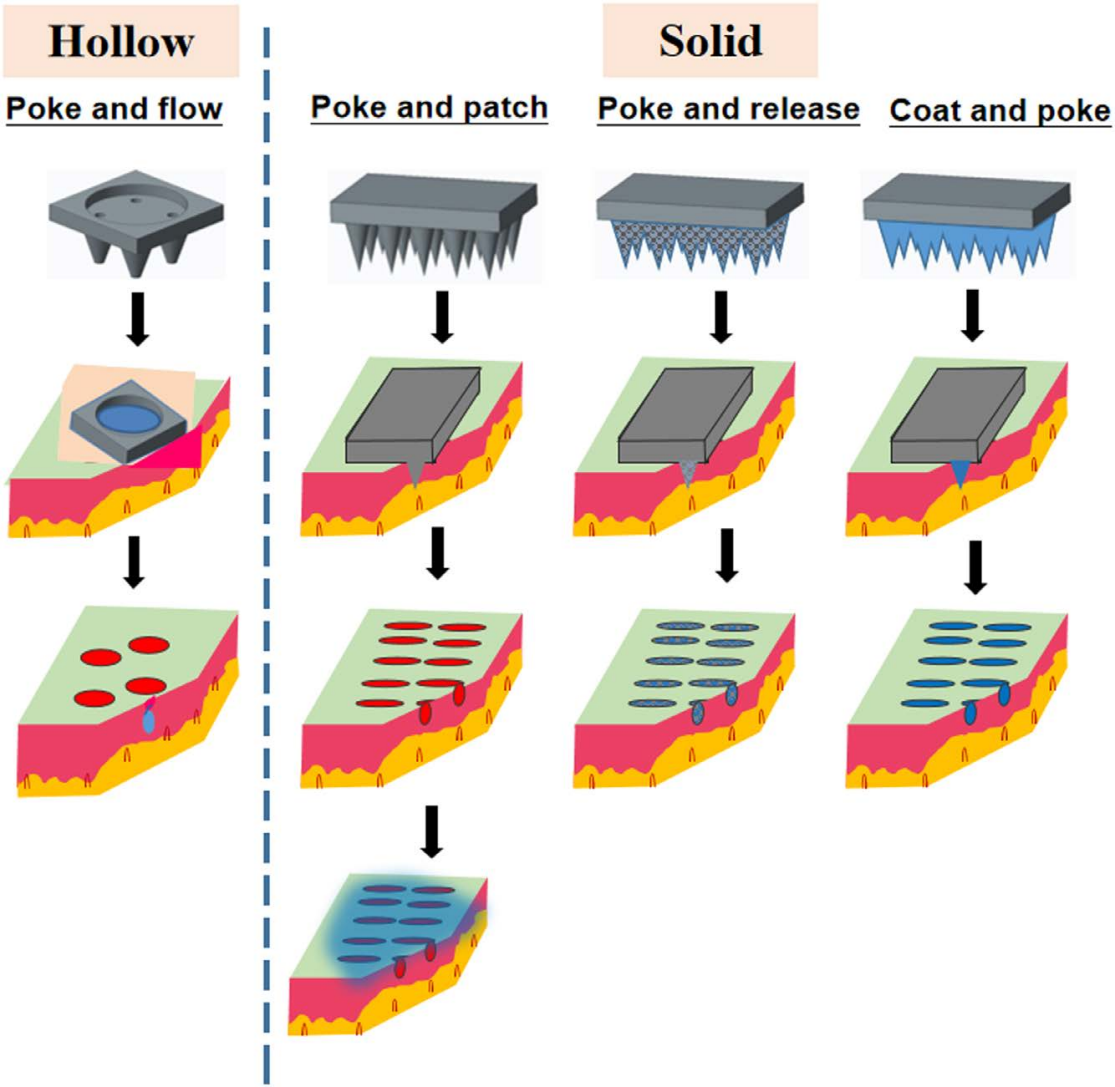
Schematic illustration of hollow and solid microneedles.

MNs are commonly used to minimally disrupt the outermost layer of the skin to enhance transdermal drug delivery. MN devices rely on a large number of micron-level needles to penetrate the epidermal layer of the skin to achieve skin penetration. MNs can be divided into hollow and solid MNs according to their structure (Fig. 1). According to the type of materials, MNs can also be divided into metal and soluble polymer MNs. MNs promote transdermal drug penetration and accumulation in specific sites by piercing into the skin to provide permeation channels.^[34]^ MNs are suitable for many drugs, including macromolecules, such as peptides, proteins, small interfering RNAs, oligonucleotides, and vaccines. However, the disadvantages of MNs are that they can deliver only a limited drug dose, and they have insufficient mechanical strength. Further research is required to overcome these disadvantages.

Hollow MNs pierce the skin and release the drug solution through the internal pores, which is similar to subcutaneous injection.^[35]^ Hollow MNs bypass the SC to directly deposit the drug into the live epidermis or dermis in a manner that is particularly suitable for high MW drugs.^[36]^ Hollow MNs consist of a catheter structure, particularly for a fluid or liquid formulation, driven by the external pressure, which can be adjusted to vary the drug delivery speed.^[37]^

Solid MNs are commonly used in the “poke, separate, and diffuse” format and can be applied on the skin surface to destroy the SC physically. Removal of solid MNs from the skin results in a brief microchannel in the SC, after which the pharmaceutical preparation can be applied, such as solution, suspension, gel, cream, or patch. The delivery of drug formulations penetrating into those microchannels mainly depends on passive diffusion. Systemic delivery or local effects of therapeutics depend on absorption first and then on transportation. Solid MNs can increase the transdermal permeability of nanoparticles by up to 4 times.^[38]^

MN rollers are also a method of improving drug delivery efficiency. An MN roller is a cylindrical stainless-steel MN that is rolled over the surface of the skin to create micropores. It could improve transdermal delivery of antiallergic drugs through simulation experiments using pig skin. Commercial formulations combined with MNs are a relatively mature technology.

The hydrogel MNs containing donepezil hydrochloride were prepared and applied to pig skin. At 24 hours after the MNs were removed, donepezil hydrochloride had a higher in vitro permeability and plasma concentration, demonstrating its success delivery.^[39]^

During drug/vaccine loading, the bioinspired use of biomimetic methods can help orient fluid transport. This design will benefit the drug/vaccine loading process on the needle surface by improving efficiency and reducing drug/vaccine waste during the MN coating process.^[40]^

Capsaicin-loaded dissolving MNs (DMNs) were prepared to investigate the analgesic effect of capsaicin on skin. The dimensions of each MN were as follows: diameter of the base, 17 mm; length, 500 μm; and width, 300 μm. The average capsaicin content in the DMNs loaded with a low and high dose of capsaicin was 8.8 ± 0.5 and 12.5 ± 0.4 mg, respectively. Almost all the capsaicin, 99.3 ± 4.1% and 99.7 ± 2.2% for low-dose and high-dose DMNs, respectively, were released within 20 minutes. The pharmacological activity of capsaicin DMNs was compared with that of capsaicin cream as a positive control, by measuring the idiospasm of depilated rat skin. The time required to achieve 50% idiospasm suppression was 26.3 ± 1.9 and 53.0 ± 2.3 minutes for low-dose and high-dose DMNs, respectively. The different loading of capsaicin leads to the difference in the time to suppress spasticity. A pharmacokinetic study showed high tissue capsaicin levels of 660.2 ± 120.6 and 1805.3 ± 218.1 μg/g wet weight for low-dose and high-dose DMNs, respectively, at 5 minutes after administration. The results suggested that DMNs could exert a rapid local analgesic action on the skin.^[41]^

Compared with the traditional H1N1 influenza vaccine microneedles with stabilizers, the stabilizer-free H1N1 microneedles prepared in a 10 °C production environment induced the obvious protective immune response. In animal experiments, mice showed a strong antibody response with an array of low-temperature H1N1 microneedles without a stabilizer (LT-MN). Compared with the traditional intramuscular immunization, low-temperature H1N1 microneedles with a stabilizer (LT-MN-T), and room-temperature H1N1 microneedles with a stabilizer (RT-MN-T), LT-MN produced comparable results in inducing protective immunity. A plaque reduction neutralization test found that LT-MN and LT-MN-T provided greater immunity compared with intramuscular immunization and RT-MN-T. This stabilizer-free low temperature H1N1 microneedles are suitable for a variety of temperature-sensitive biological agents.^[42]^

### 4.2. Lasers

**Figure 2.**
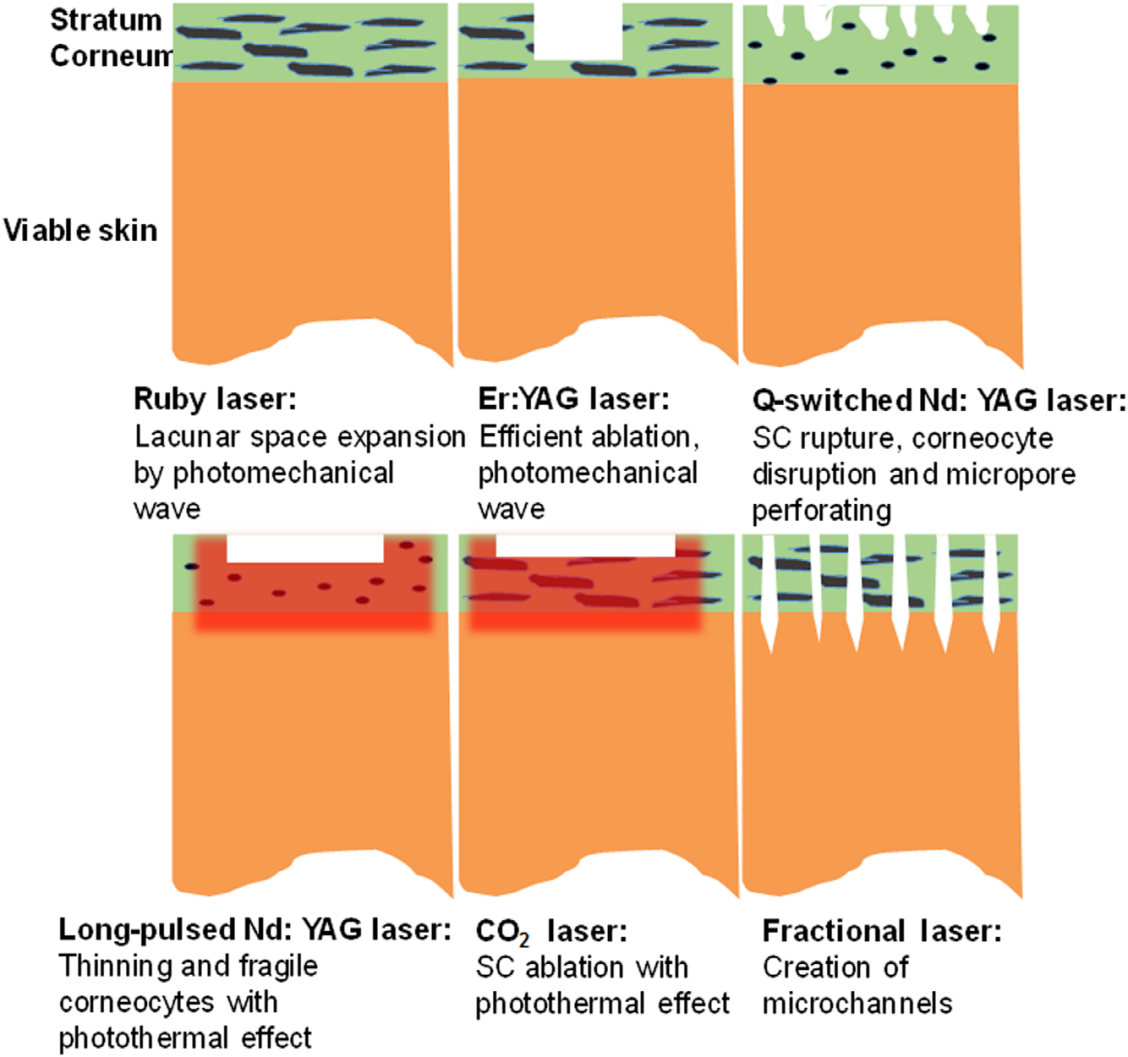
Overview of the different mechanisms of laser-facilitated drug delivery. SC = stratum corneum.

Lasers are an important way to increase transdermal permeability (Fig. 2). Lasers can ablate the SC and thus enhance drug delivery. Laser-induced skin damage recovers quickly after several days. Lasers can directly damage the skin barrier through the photothermal effect to increase the transdermal absorption of drugs. This method can increase bioavailability and shorten the treatment period.^[43]^ The transdermal effects of various drugs can be enhanced using lasers.^[44,45]^ Despite the low energy output of the lasers used, laser safety remains a controversial issue.^[46]^ However, one of the problems that limits laser application is that specialized laser equipment is very large and not appropriate for personal use.

Laser irradiation can interact with skin tissues, such as SC, epidermis, and dermis. Skin is a semipermeable barrier that protects the body from external damage and prevents moisture loss.^[47]^ Laser has not only reduced the topical dose required but also allows drugs to penetrate deeper into the soft tissues.

Different types of lasers have different wavelengths and mechanisms, including ruby (694 nm), yttrium-scandium-gallium-garnet (2790 nm), erbium: yttrium-gallium-garnet (Er:YAG, 2940 nm), neodymium-doped yttrium-gallium-garnet (Nd:YAG, 355, 532, 1064, and 1320 nm), and CO_2_ (10600 nm) lasers.

The ruby laser emits laser light at 694 nm and is used in clinical dermatology to treat freckles, pigmented skin lesions, macula, and chloasma.^[48]^ This laser can produce optomechanical waves that facilitate local/transdermal drug administration. The application of optomechanical waves occurs in a very short time of 10 ns-1 μs. The permeabilized photomechanical wave on the SC is temporary and returns to normal after a few minutes of irradiation.^[49]^ The penetration of photomechanical wave-assisted dextran (5 J/cm^2^, 490 ns) reached 400 μm into the rat dermis. Extensive and sustained distribution of dextran in live skin indicated that the delivery route was intercellular or intracellular. Another study investigated the application of a single photomechanical wave from ruby laser to rat skin for the local delivery of nanoparticles of about 100 nm. Exposure of skin to the photomechanical wave allowed the nanoparticles to diffuse into the epidermis. This demonstrated that the photomechanical wave can promote the local delivery of very large permeates.^[43]^

An Er:YAG laser emits light with a wavelength of 2940 nm and is used as a surface repair tool for microdermabrasion, in which the emitted light is effectively absorbed by water molecules. Its shallow radiation into the skin can precisely ablate the SC to reduce thermal damage. Er:YAG lasers used for ablation have a shorter healing time, induce less erythema, and cause fewer pigmentation problems.^[50]^

The use of an Nd:YAG laser is another resurfacing technique with skin ablation ability that facilitates drug delivery. Nd:YAG lasers are effective for dermatological treatment and cosmetic regeneration in clinical applications, such as pigmentation, scar repair, hair removal, skin remodeling, and lipolysis.^[51]^ Nd:YAG lasers with a long pulse (LP, 15 J/cm^2^) and Q-switched (0.5 J/cm^2^) lasers were evaluated for their ability to improve glycerol transdermal delivery in rats.^[52]^ Lasers delivering an LP could attenuate SC barriers, which may be adversely affected by SC interruption caused by the photothermal effects. The QS laser destroyed the SC, possibly because of its high energy output and the enormous pressure in the ultrashort range. This enhancement effect may last for at least 1 week without infection.

CO_2_ lasers have an emission wavelength of 10,600 nm. They produce instantaneous heating of water and cause tissue vaporization. The residual thermal effect causes coagulated skin and necrosis. CO_2_ lasers usually ablate the SC, and the skin thickness is thinner than that resulting from ER:YAG treatment. In addition, the thermal effect can rejuvenate the skin for a cosmetic effect after optimizing the viable laser energy. Transdermal delivery of 2 vitamin C derivatives, 3-O-ethyl ascorbic acid and ascorbic acid 2-glucoside, with or without Er:YAG and CO_2_ lasers, was compared. The results showed that the permeability of the 2 vitamin C derivatives pretreated with Er: YAG or CO_2_ lasers increased markedly.^[53]^

Three vitamin C derivatives were used as model drugs, and the transdermal ability of 2 Er:YAG and CO_2_ lasers was compared. The results showed that the transdermal volume of all 3 drugs increased by several tens of times. This showed that these 2 lasers have a significant effect on increasing the transdermal absorption of drugs.^[54]^

Studies have shown that 1064–nm Nd:YAG lasers with an LP (15 J/cm^2^) can loosen keratin and make the keratinocytes brittle or detached, whereas the output mode of Q-switched (0.5 J/cm^2^) completely destroys keratin or keratin cells, forming perforations on the SC. It was concluded that the degree of enhanced transdermal delivery is different for different output modes.^[55]^

Currently, nanotherapy combined with stimulation-activated activators has become a focus of research attention. For example, Fe_3_O_4_ nanoparticles were used as the core of a drug-loaded hydrogel coating, and folic acid was grafted onto the surface of the composite material to construct laser-sensitive magnetic nanoparticles. This method could not only perform targeted therapy but also improved the transdermal absorption of the drug, thereby improving the effect of the drug to treat the disease.^[56]^

During a scalp test using a fractional Er:YAG laser, it was found that the transmitted pulse energy might affect the size and/or depth of the microchannels created by the laser, and the duration of the pulse might affect the amount of thermally changed tissue. This meant that the tissue ablation threshold was lowered with shorter pulse durations and higher power. For smaller molecules, transdermal delivery efficiency is increased by increasing the size of the microchannels created by the laser and can partition easily into the skin when less thermal damage occurs. In comparison, the transportation of larger molecules through the skin becomes increasingly difficult.^[44]^

### 4.3. Iontophoresis

Iontophoresis is the application of a small electrical current (< 5 mA/cm^2^) to increase the transdermal penetration of a drug, especially the dissociation of drugs into or through the skin.^[57]^ The transdermal enhancement mechanism of iontophoresis depends on electric current, which leads to differences of potential and concentration.^[58]^

The advantages of iontophoresis-assisted transdermal delivery include easy application, safety, high transdermal efficiency, and miniaturized instruments. Limitations include the limited application range and longer operation time. If iontophoresis could be applied for peptides, proteins, and other macromolecules, they would have a promising and wider application.

**Figure 3.**
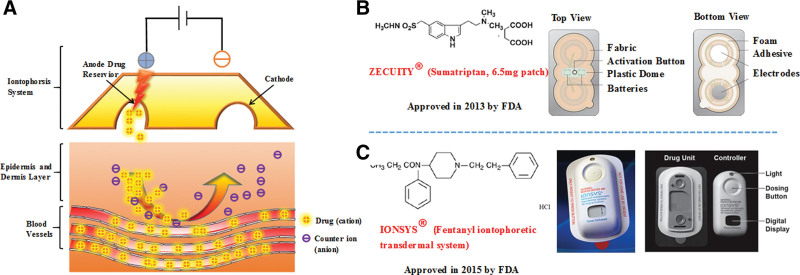
Iontophoresis for transdermal permeation. (A) The principle of iontophoresis using an Ag/AgCl electrode system. (B), (C) Commercial formulations using iontophoresis. A^−^ = anionic drugs, Cl^−^ = chloride ions in the drug reservoir, D^+^ = cationic drugs, FDA = Food and Drug Administration, Na^+^ = sodium ions in the drug reservoir.

Currently, there are 2 commercial formulations composed of iontophoresis (Fig. 3). The sumatriptan iontophoresis system was approved by Food and Drug Administration (FDA) for the acute treatment of adult migraine in 2013. The transdermal system delivers sumatriptan with a low level of current and bypasses the gastrointestinal tract. Pharmacokinetic studies indicated that iontophoretic delivery of sumatriptan reached a maximum serum concentration of 22 ng/mL at 15 minutes after activation. A linear relationship was found between the total applied current and the sumatriptan delivery ratio. Patches with 6 and 12 mA per hour produced a favorable plasma level of sumatriptan that exceeded 10 ng/mL for more than 7 hours. In addition, patients with the sumatriptan patch had a painless rate of 18% at 2 hours after administration with iontophoresis compared with only 9% (*P* = 0.0092) for the placebo. Oral sumatriptan may cause severe nausea or vomiting for migraine patients. This sumatriptan transdermal system may be a very good choice to avoid such adverse events.^[59,60]^

Estradiol-loaded nanoparticles combined with iontophoresis can significantly increase the permeability of estradiol, which is advantageous for the treatment of osteoporosis. Iontophoresis facilitated the permeation of nanoparticles through follicles into capillary vessel and finally reached the effective blood concentration. The bone mineral density with iontophoresis was significantly higher than that of the passive diffusion and the control groups.^[61]^

Postoperative pain is usually controlled by patient-controlled analgesia.^[62]^ The use of noninvasive transdermal analgesics (such as lidocaine, fentanyl, and morphine) overcomes the problems associated with patient-controlled analgesia. For example, iontophoresis can drive fentanyl molecules through the skin into the systemic circulation.^[63]^ The fentanyl iontophoretic transdermal system is generally effective in the well-tolerated acute postoperative management of hospitalized patients with severe pain in the abdomen and chest, or in orthopedics. Overall, fentanyl iontophoretic transdermal system provides the same analgesic effect compared to morphine but is considered more convenient and easier for patients to use.^[64]^

Transdermal enhancement by iontophoresis on the penetration of anti-inflammation drugs was studied. Iontophoresis could disrupt the arrangement of lipids between cells and increase their fluidity. Moreover, the intercellular space is enlarged, and the SC structure is loosened, which promotes transdermal administration. In addition, the degree of drug dissociation has a significant effect on the enhancement by iontophoresis. The higher the degree of drug dissociation, the more obvious the transdermal enhancement effect.^[65]^ The effects of various technologies on the transdermal delivery of 3-fluoroamphetamine hydrochloride were compared, and the results showed that iontophoresis had the most significant effect in increasing the transdermal effect of the drug. Thus, iontophoresis was considered superior to the other transdermal enhancement techniques.^[66]^

Experiments have shown that neurotensin-loaded silk fibroin films not only release neurotensin quickly to regulate inflammation after being stimulated by iontophoresis but also that anode iontophoresis has a certain inhibitory effect on gram-positive bacteria. Thus, stimulation of peptide fibrin membranes by iontophoresis has broad prospects for treating wounds.^[67]^ A study used the prodrug ketoprofen choline chloride as a model drug for nonsteroidal anti-inflammatory drugs. After treatment with iontophoresis, the transdermal delivery rate of ketoprofen choline chloride could be increased by several times.^[68]^ Another study has proved that the permeability of nanoparticles treated with iontophoresis is higher than that of pure nanoparticles.^[69]^

### 4.4. Sonophoresis

Sonophoresis is a physical technique that uses ultrasound (US) to promote transdermal absorption. The advantages of sonophoresis include its noninvasive nature, little damage to the skin, simple operation, high universality, and suitability for almost all drugs. However, sonophoresis requires an ultrasonic coupling agent, commonly hydrogels, to transmit US. Therefore, active chemical agents could be added to the ultrasonic coupling agent, which could achieve physical therapy and transdermal drug delivery simultaneously.

Current research suggests that the mechanism of increased transdermal delivery by sonophoresis mainly includes a cavitation effect, thermal effect, and mechanical effect.

#### 4.4.1. Cavitation effect.

When the ultrasonic wave propagates in the medium, the average distance of the molecules varies with the molecular vibration. When it exceeds the critical molecular distance, a cavitation effect is formed.^[70]^ Cavitation can instantly produce high temperature and pressure, accompanied by strong shock wave or ray flow, resulting in disorder of the lipid bilayer structure in the SC. The cavitation effect is considered as the main mechanism by which sonophoresis facilitates transdermal drug delivery.

#### 4.4.2. Thermal effect.

The thermal effect refers to the phenomenon that the medium absorbs energy that is attenuated by the ultrasonic wave in the propagation process, which causes increased temperature.^[71]^ The higher the temperature, the higher the molecular diffusion rate. Therefore, rates of blood flow and drug dissolution are accelerated. This is advantageous for drug permeation.

#### 4.4.3. Mechanical effects.

**Figure 4.**
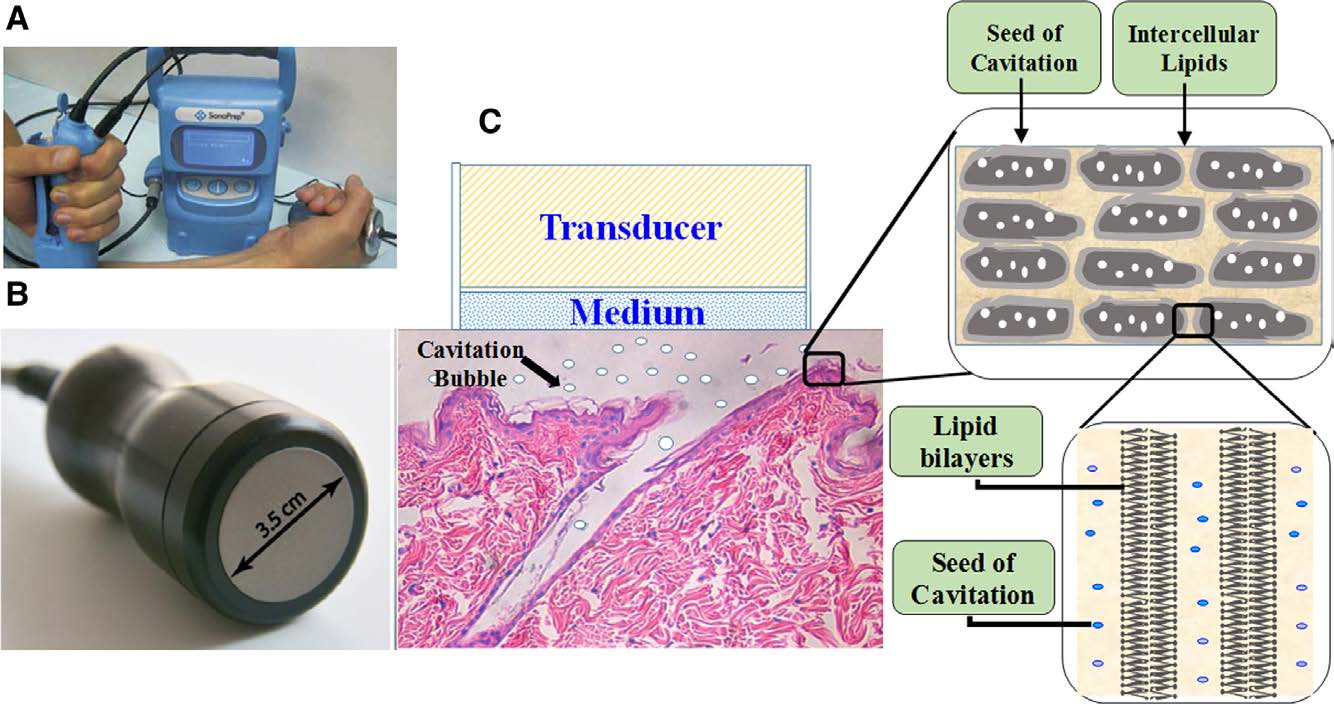
Sonophoresis for transdermal permeation. (A) Low-frequency ultrasound treatment in a clinical setting with the SonoPrep® device (Echo Therapeutics, Franklin, MA). (B) Ultrasound transducer, 3.5-cm diameter. (C) Mechanistic overview of sonophoresis-facilitated drug delivery

US can transfer energy through mechanical vibration. During high-speed ultrasonic vibration, the structure of the lipid layer in the SC changes, and its permeability increases. When the mechanical intensity is high enough, pores in the cell membrane form, and thus, the permeability of the drug increases. This effect is also called the “acoustic pore effect”^[72]^ (Fig. 4).

It is generally believed that frequencies of 20–100 kHz comprise low-frequency sonophoresis (LFS), and frequencies of 0.7–16 MHz comprise high-frequency sonophoresis (HFS).^[73]^ According to previous research, LFS seems to be more effective in increasing skin permeability and delivering a wide range of molecules and formulations, such as proteins, biopolymers, and vaccines, compared with that of HFS.^[74–76]^

Transient cavitation is the most important mechanism by which LFS enhances skin permeability. The enhanced skin permeability by cavitation activity on the skin surface was evaluated using porous resin as a cavitation nuclear coupling medium.^[77]^ The microjets produced by LFS could penetrate into the skin or collapsed near the skin surface to cause structure disorders. In addition, the collision of the cavitation foam on the surface had an important effect on enhanced skin permeability.

Cavities may play an important role in transdermal delivery by HFS.^[78]^ Histological examination revealed that voids are formed in the skin after an HFS of 10 and 16 MHz. The voids in the keratinocytes led to direct interaction with the oscillating cavitation bubbles, which induced a disordered lipid bilayer and increased skin permeability.

Some studies have used ketoprofen as a model drug and found that both poly(amidoamine) dendrimers and US treatment could increase the transdermal absorption of the drug, but combining the 2 further improved the transdermal ability of ketoprofen.^[79,80]^ Experiments have compared the transdermal effects of dual-frequency US (20 kHz + 1 MHz) and single-frequency US (20 kHz), and found that dual-frequency US could expand the pores to increase the permeability of the drug to a greater extent, while reducing the treatment time, without causing increased damage to the skin.^[81]^ Over a decade ago, LFS was combined with chemical penetration enhancers (CPE) for the first time and was found to have a synergistic effect in increasing the permeability of the skin to drugs.^[82]^

### 4.5. Electroporation

EP is a technique used to increase the permeability of the cell membrane. Nowadays, it is used to promote drug transdermal or transmucosal absorption. EP uses an instantaneous high-voltage pulsed electric field to form a temporary, reversible hydrophilic channel in a lipid bilayer of the cell membrane. The size and duration of the channels are related to the voltage, pulse number, and pulse time.^[83]^ Many studies have indirectly proposed the formation of “holes” or “water channels” formed via EP. These holes are small (<10 nm), temporary, and sparse (about 0.1% of the surface area).

The advantages of EP are its versatility. First, it can strictly control the transdermal permeation rate. Second, the application range of EP is wide, being especially suitable for biomacromolecules delivery, such as peptides, proteins, and vaccines. Consequently, there is no selectivity for drugs and the other therapeutic agents through skin using EP.

**Figure 5.**
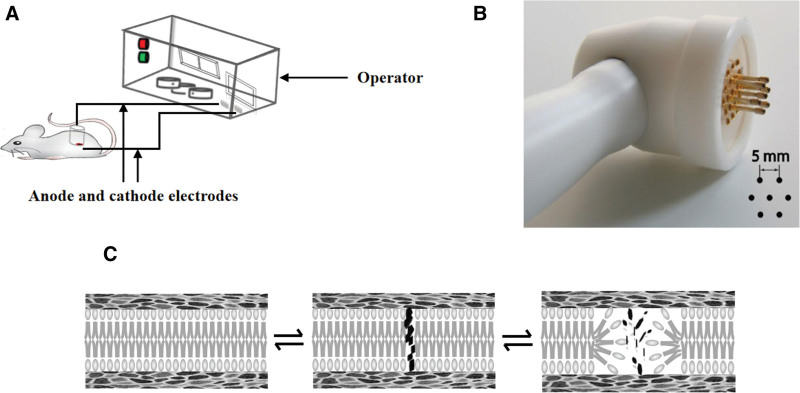
Electroporation for transdermal permeation. (A) The components of an electroporation device. (B) Electroporation pin electrodes arranged in a honeycomb configuration. (C) Mechanistic overview of electroporation facilitated drug delivery.

Transdermal EP occurs when the skin is exposed to high electric field pulses, also known as electroosmosis.^[84]^ EP is a physical transfer mechanism that applies electrical impulses directly to cells or tissues, which transiently permeabilizes the plasma membrane, increasing the permeability of mammalian cell membranes and allowing macromolecules to enter cells, thereby effectively delivering exogenous molecules.^[85]^ According to the hole formation theory of water thermodynamics, water molecules penetrate into the lipid bilayer of the membrane, resulting in rearrangement of adjacent lipids and their polar head groups. The EP mechanisms include the following steps. First, a complete phospholipid bilayer before the EP is required. Second, because of the interaction between the water dipoles and the electric field after EP, changes in the ultrastructure of SC lipids cause water molecules to penetrate the bilayer to form a water filament, and the ingress of water increases the likelihood of pore formation, resulting in the formation of many pores per unit area and per unit time in the phospholipid bilayer. Finally, the lipids adjacent to the water begin to be realigned with their polar heads, stabilizing the pores and allowing more water and other polar molecules to enter (Fig. 5).

Low-dose cisplatin delivered by EP was evaluated as a new treatment strategy for osteosarcoma on 2 osteosarcoma cell lines.^[86]^ The results indicated that the metabolic regulators delivered by EP were more effective in inhibiting the cell cycle of osteosarcoma cells and had a negative impact on their ability to recover and proliferate.^[86]^

Arthritis in the right knee joint of rats was established to evaluate the anti-inflammatory and analgesic effects of diclofenac sodium hydrogel delivered via EP. EP was applied using a high-voltage pulse of 900 V for 8 minutes with 5 ms on and 20 ms off. The results indicated that EP significantly increased the plasma concentration of diclofenac. The therapeutic benefit provided by EP was comparable with that of oral diclofenac.^[87]^

Cisplatin EP toxicity toward human pancreatic ductal adenocarcinoma cell lungs in vitro showed that cisplatin EP has a higher cytostatic effect than cisplatin alone.^[88]^ Studies have shown that EP and reverse iontophoresis have synergistic effects in promoting skin penetration.^[89]^

A new EP method that could effectively deliver DNA and small interfering RNA was proposed. Nucleic acid molecules could be delivered through mouse skin by combining MNs and flexible interdigitated electroporation arrays. This technology has laid the foundation for dermatological or intradermal vaccination.^[90]^

### 4.6. Magnetophoresis

**Figure 6.**
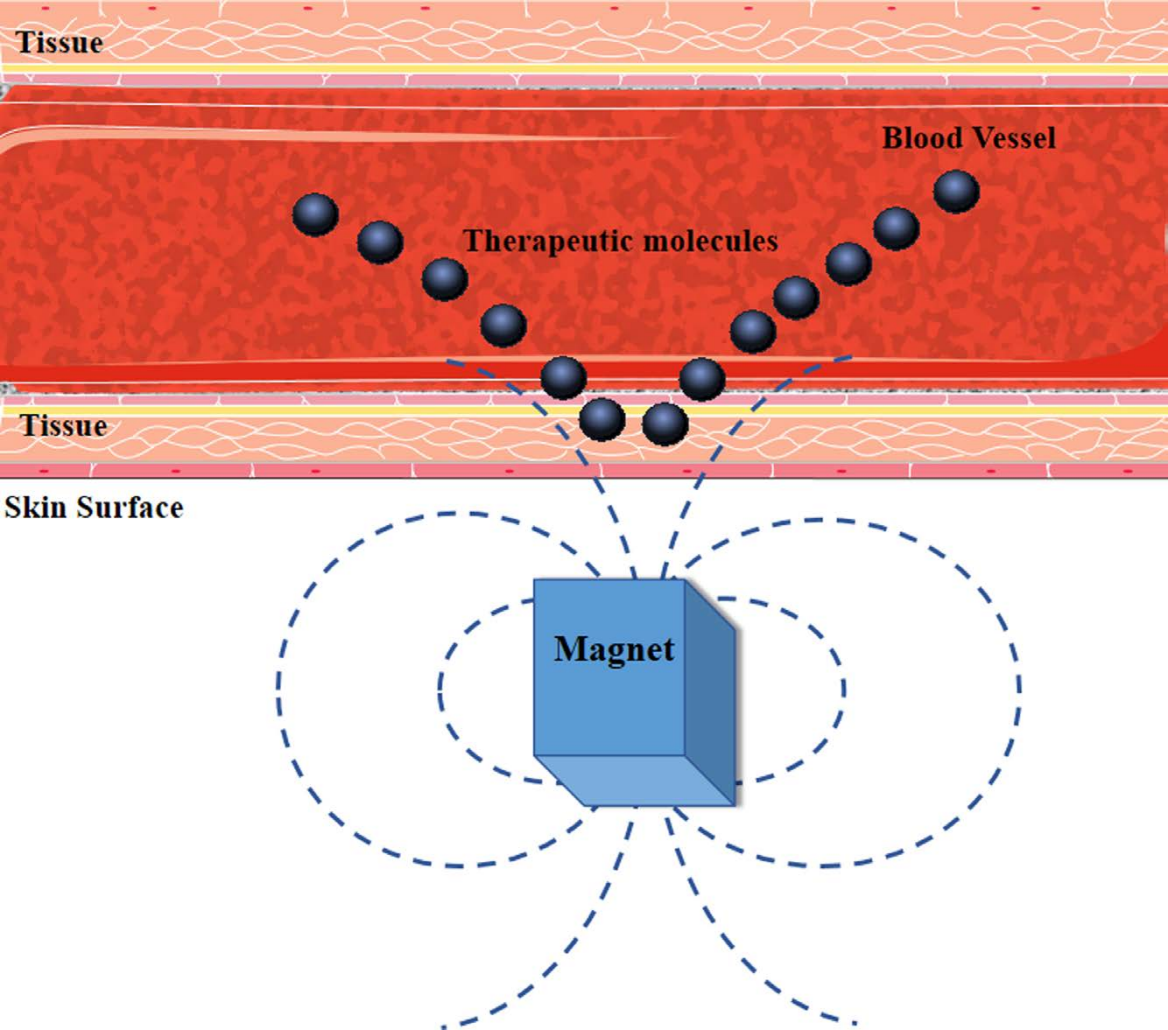
Schematic representation of a magnetic nanoparticle-based drug delivery system. The steps for magnetic NP-based drug delivery are: Coupling the drug to the MNP; applying an external magnetic field to attract the MNP to the desired location, such as a tumor; and the release of the drugs from the NP under the influence of an external alternating magnetic field once it reaches the target. MNP = magnetic nanoparticle, NP = nanoparticle.

There are few studies of magnetophoresis in transdermal delivery. Magnetophoresis works by applying a magnetic field to enhance the passage of drugs across a biological barrier. The stronger the magnetic field, the better the transdermal effect (Fig. 6).^[91]^ The fixed magnetic field could increase permeation flux enhancement factor, and the alternating magnetic field could enhance drug penetration. Both of the combined use has a massage-like effect on skin. The fluorescence signal was detected in deeper regions of the skin with a larger area with the combination application of the stationary and the alternating magnetic fields.^[92]^

### 4.7. Microwave-assisted transdermal delivery

In recent years, microwave technology has received widespread attention in the transdermal field. Microwave technology promotes transdermal drug delivery by allowing the lipid structure of the SC to enter the nondomain.^[28,93]^ In addition, microwaves have a certain synergistic effect with CPEs.^[94]^

A study combined chitosan nanoparticles with microwave technology. Microwave and chitosan nanoparticles affected the ceramide, palmitic acid, and keratin domains of the skin, thus achieving synergistic promotion of transdermal administration.^[95]^

Microwaves are considered one of the most promising transdermal delivery technologies. However, further research is needed to explore their value in the medical field.

### 4.8. Combined technologies to improve topical delivery

#### 4.8.1. Iontophoresis and chemical enhancers.

Both CPEs and iontophoresis have good transdermal promotion effects. Thus, experiments have combined both techniques, with lidocaine HCl, nicotine hydrogen tartrate, and diltiazem HCl as model drugs. The in vitro percutaneous treatment of each drug in pig skin and buccal tissue was compared, together with enhanced buccal delivery. The results demonstrated that the enhancement of transdermal delivery by iontophoresis was more pronounced than the enhancement achieved by buccal delivery of the drugs.^[96]^

#### 4.8.2. Lasers and microneedles.

There is an urgent need to substitute noninvasive or minimally invasive delivery methods for the injections currently used for low MW heparin multidose therapy. In a study, a laser-engineered dissolution MN (DMN) array was fabricated. This technique does not reduce the strength of the MN or the biological activity of the drug. This method is not only a relatively low-cost functional delivery system but also has great potential for the transdermal delivery of large molecules.^[97]^ A study showed that silica-coated lanthanum hexaboride (LaB6 @ SiO_2_) nanostructures could be incorporated into polycaprolactone MNs as infrared absorbers. Under infrared light, the light and heat generated by the infrared absorbers were transmitted to cause the MNs to melt, thereby releasing the drug. This near-infrared, light-triggerable device has excellent reproducibility, low state leakage, and noninvasive trigger ability, and thus represents an advance in transdermal drug delivery technology.^[98]^

#### 4.8.3. Microneedles with electroporation and sonophoresis.

MNs, EP, and US therapy are 3 transdermal methods for physically enhancing drug delivery. Some studies have used fluorescein isothiocyanate-dextran as a model drug and studied combinations of the 3 technologies (i.e., microneedle–electroporation [MN-EP], microneedle–ultrasound, electroporation-ultrasound [EP-US], and microneedle–electroporation–ultrasound [MN-EP-US]) for skin penetration in vitro. The experimental results showed that the combination of all 3 technologies (MN-EP-US) caused the most significant increase in the permeability of fluorescein isothiocyanate-dextran.^[99]^ The permeability of bovine serum albumin increased to 1 μm/s with the microneedles patch combined with 15-W US output, which is about 10 times higher than the that of the passive diffusion. This technique provides the possibility of transdermal absorption of large molecules with high efficiency.^[100]^

#### 4.8.4. 3D printing.

3D printing technology appeared in the mid-1990s. First, a blueprint is drawn by a computer according to demand; then, the “printing material” is superimposed layer by layer using technologies such as light curing and paper lamination under computer control. Finally, the blueprint on the computer becomes a physical object. Here, we review 3D printing in transdermal drug delivery.

As a technology precisely controlled by a computer, 3D printing can print a transdermal patch that perfectly fits the human skin according to the precise and complete contours of the human body, which could increase the transdermal delivery rate of the drug by improving its bioavailability. Researchers obtained a 3D computer-aided design model of human volunteers’ heads and faces through a handheld 3D scanner and then produced a proprietary nasal patch using 3D printing.^[101]^ The use of specific nose patches that fitted the patient’s skin perfectly provided a more accurate dose of the medicine.

One of the important applications of 3D printing in transdermal delivery is MN fabrication, which can create micropores in the skin through a large number of micron-sized needle tips, thereby allowing macromolecular drugs to penetrate the skin, such as insulin^[102]^ and melanostatin.^[103]^ MNs on a personalized curved surface meant that each MN is perpendicular to the skin surface and can fully insert into the skin. The stereolithography 3D-printing technique was used to fabricate the hollow MNs interfaced with the microfluidic structures. Compared with the previous methods, the method was with lower cost, higher printing speed, and higher throughput.^[104]^

**Figure 7.**
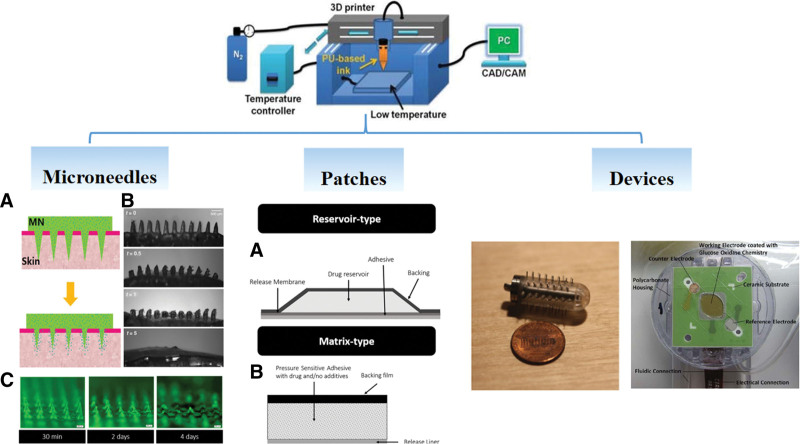
3D printing in transdermal delivery. CAD = computer-aided design, CAM = computer aided manufacturing, MN = microneedle, PC = personal computer.

3D printing allows rapid optimization of materials and changes to the geometry to fabricate personalized MNs. In addition, the advantages of 3D printing in transdermal delivery are represented by personalized electrical devices (Fig. 7).

## 5. Conclusion

**Figure 8.**
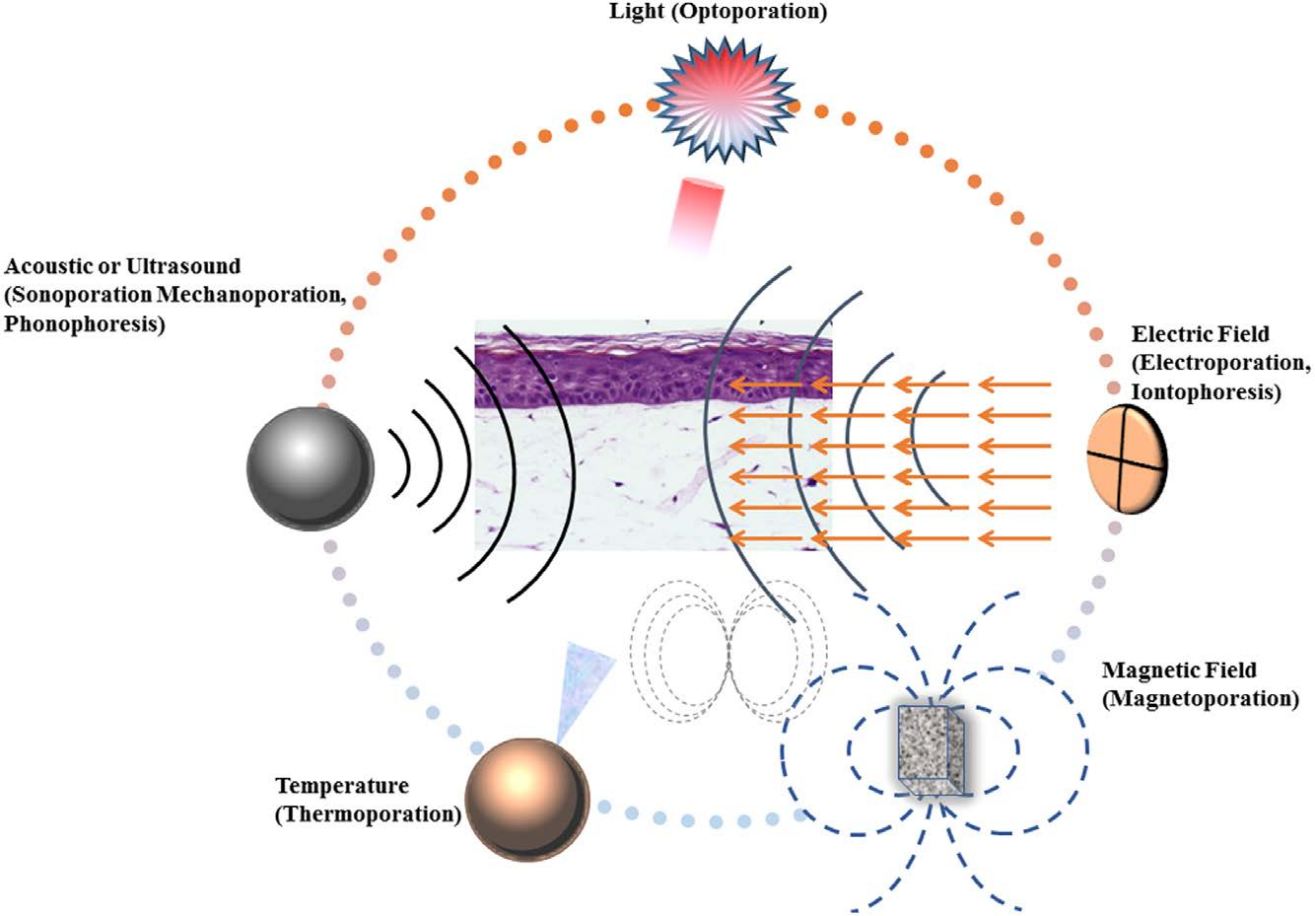
Different physical techniques to improve transdermal delivery.

TDDSs have many advantages; therefore, they have attracted more and more attention in the field of medicine, especially from pharmaceutical companies. There are more than 20 FDA-approved transdermal drugs according to the published reports. Most of these drugs have been delivered via other administration routes before being used for transdermal drug delivery, emphasizing the growing interest in transdermal administration. Most drugs currently approved for transdermal administration rely on passive administration or moderate levels of permeability. However, the multiple physical techniques may be highly effective to improve transdermal efficiency (Fig. 8). Applying these technologies to the preparation of miniaturized wearable electronic devices has broad commercial prospects. The advantages and disadvantages of different physical technologies are diverse. Overall, the properties of a drug determine which is the best method to improve its transdermal efficiency, such as MW, pKa, and logP.

About half of the currently available commercial products have the potential to be tested as a vaccine, and many vaccines are currently undergoing clinical trials for various incurable diseases, such as various cancers and HIV. Some transdermal techniques can also be applied to vaccination of any disease, and as research continues, the goal of vaccine self-management could be achieved. The use of live vectors that can trigger an immune response in current viral vaccines for injections has limited the ability to immunize to some extent. However, the use of vaccines that apply EP for DNA is now feasible. This has the potential to achieve immunization against various cancers and viruses. MN technology also has potential in this respect. These attractive features will continue to attract research and commercial interest.

Electrical-based drug delivery may be more restricted. Like LFS, they require electrical input and are more complex and expensive compared with MN technology, making them more difficult to implement than MN. In addition, during treatment, both EP and iontophoresis can cause discomfort or pain to the patient, especially iontophoresis. Finally, it may take several hours for the drug to reach the effective site, which makes the kinetics of drug delivery clinically meaningless. Until these devices are further optimized, the combination of the 2 methods will only complicate the treatment plan.

The key advantages of physical technologies are their wide application for previously nonappropriate transdermal drugs, such as proteins, peptides, and hydrophilic drugs. Based on the improved permeation of drugs via multiple physical techniques, many more diseases and conditions may be treated, such as tumors, dementia, Parkinson’s disease, chemotherapy-related nausea/vomiting, urinary incontinence, pain and opioid dependence, contraception, hypertension, and motion sickness. The selection depends on the advantages, disadvantages, and characteristics of the specialized permeation technologies (Table 3).

**Table 3:** Comparison of different physical technologies used for drug delivery.

Physical energy	Electric field	Magnetic field	Temperature	Ultrasound	Light
Poration	Electroporation	MNP	Thermoporation	Sonoporation	Optoporation
Limitations	Narrow range of clinically safe electric field parameters (refer to current standards for safety levels)	Limited drug carrying capacity of magnetic field due to their biodistribution	Low penetration depth (since only applied topically so far)	Sonoporation devices have poor calibration in terms of the amount of ultrasound energy emitted	Limited time duration between optoporation and drug delivery
		Narrow range of magnetic field			
Disadvantages	Irreversible electroporation, cell death with high fields	Aggregation of MNP can cause embolization	Excess heat can induce thermohemolysis	Shear forces may induce rupture of cells	Excessive inflammation, postinflammatory, and hyperpigmentation
	Electromechanical coupling effect	Cytotoxicity increases with the increased concentrations of MNP	Relies on electric field to heat up the filaments; therefore, the disadvantages of electric field applies	Temperature increases as a function of frequency and eventually disrupts cells	Laser usage and ultrastructural changes in epidermis
Advantages	Inexpensive and simple to perform	Noninvasive nature of the magnetic field	Noninvasive nature of low heat compared to EP	Less invasive compared to EP	Remote operation with less cellular damage
	Drugs are easy to overcome the cell membrane barrier	Field modulated externally without electrode contacts unlike EP	Selective irreparable cellular damage	Instant impermeabilization after ultrasound exposure	Enhanced optofection efficiency compared with regular gene delivery
		High efficiency of drugs delivery compared with that without the magnetic field			Deep penetration; key nanosurgical tool to the microscopist

EP = electroporation, MNP = magnetoporation.

However, the extensive application of the physical technologies combined with therapeutic agents is dependent on the related convenient and portable devices. Most of the traditional transdermal technologies are assisted by equipment. In addition to improving the equipment, more new technologies such as MNs should be explored. In the development of the device, while blindly increasing the permeability of the drug, it should be considered that the doctor and patient can use it together and take advantage of transdermal drug delivery.

Combined products comprising medicine and devices are future commercial directions associated with artificial intelligence and 3D printing. Further research should break through the limitations of traditional transdermal drugs and explore more drug potential for transdermal absorption.

As a newly developed drug delivery system, the TDDS has its unique advantages over other drug delivery methods. However, there is still much room for development and implementation of TDDSs. Research directed at unmet clinical needs will be particularly promoted by the benefits provided by specific methods, which will drive innovation. In view of its more humanized drug treatment characteristics and the continuous development of transdermal technology, it will have broader prospects in the future.

### Author contribution

Conceptualization: Yan Gao, Lina Du, Shan Ma, Xiu Wang, Bonian Zhao.

Data curation: Yan Gao, Qian Li, Qi Li.

Formal analysis: Yan Gao, Qian Li, Lin Zhu.

Investigation: Yan Gao, Lina Du.

Methodology: Yan Gao, Qian Li, Lina Du

Project administration: Meiyan Yang.

Software: Lina Du, Shan Ma, Xiu Wang.

Writing - original draft: Yan Gao, Qian Li, Lin Zhu.

Writing - review and editing: Yan Gao, Qian Li, Qi Li, Lina Du

### Acknowledgments

We are thankful for the Key Technologies for Quality Identification and Control of Traditional Chinese Medicine Formula Granules and their Industrial Application (2021CXGC010511), and Bengbu city and Bengbu Medical College Jointed Science and Technology Key Projects (BYLK201823).
